# Foreign Correspondence

**Published:** 1858-12

**Authors:** Wm. I. Arrington

**Affiliations:** Rotunda Lying-In Hospital, Dublin, Ireland


ATLANTA

Medical and Surgical Journal.

VOL. IV.] DECEMBER, 1858. [NO. IV.

### ORIGINAL COMMUNICATIONS.

### ARTICLE I.

*Foreign Correspondence.*

ROTUNDA LYING-IN HOSPITAL, DUBLIN, IRELAND, October 3rd, 1858.

**Dr. Lewis Watkins*,—DEAR SIR:—Knowing the great interest you take in everything that pertains to the "Ars Obstetrica," I send for your perusal a copy from my note book, of the following two cases of complex and difficult labour, which, from their numerous and peculiar complications, were of interest to me at the time of their occurrence, and which I think will not be entirely uninteresting to yourself.

Mrs. Murphey, aged 32, entered the Hospital 12 o'clock at night of the 14th of August in labour with first child. Early in the morning of the 15th, the waters came away, and upon an examination per vagina, the funis with the head and one hand were found presenting; an effort was immediately made to return the prolapsed cord, which failing after several ineffectual attempts, it was determined without delay to employ version as the only means of saving the child. The hand was accordingly introduced and one foot *only*, according to the rule of Dr. Simpson and other high authorities, was seized and brought down to the vagina, but here contrary to all expectation the process of delivery was completely arrested. The foot was pulled upon

* Through the courtesy of Dr. Watkins, of Franklin, Heard County, Ga., we have the opportunity of publishing the above.—EDITORS.

with all the force allowable, but to no avail, and no further progress being possible by this means, the hand was introduced into the vagina, with the view, if possible, of overcoming the obstruction by pushing up the head, which had without any conceivable reason become apparently locked with the breech at the superior trait; having failed, notwithstanding the great force employed to disengage the impacted head and cause it to ascend, and the funis in the meantime having ceased to pulsate, the welfare of the woman was alone to be considered, craniotomy as the most safe and expeditious means of completing the labour, was resorted to. The perforation was easily made through the anterior fontanelle, a fact in the operation which shows how completely flexed the body of the child must have been, and how imperfectly rotation had been accomplished.

After the extraction of the child, which still required considerable force notwithstanding the diminution of the head by the evacuation of the brain, there was discovered a deep sink or indentation on one temple just in front of the ear, this depression on the side of the head seemed to solve the whole difficulty to Drs. McClintock and Byrne, the attending accouchers, their explanation being, that the foot remaining up had become, by the act of turning, locked behind the neck or head, and that it was firmly maintained in that position by the arm of the same side passing across it, and in such manner too as to have the elbow resting on the part of the temple corresponding to the depression.

Supposing the parts of the child to have been thus disposed) and there is no more rational way of explaining the difficulty) and uterine contraction to have expanded itself, as it necessarily must, equally upon all sides, it is very easy to see why the head could not be pushed up by the hand and why it could not ascend when the foot was pulled upon. The head, foot and arm in fact were dove-tailed, as it were, into each other so that every effort at extraction by the protruding foot, so far from disengaging, actually tended to engage more intimately the impacted parts.

So much for the complication and its explanation, but the most important lesson deducible from the whole case—admitting the explanation to be the correct one—is that the rule proposed by Dr. Simpson and others and practiced in all cases of turning in this Hospital, of "bringing down one foot *only*," even when both are in reach, is at least open to serious objection, if not radically wrong. It should be recollected that the reason assigned for this *rule* of leaving up one foot, is, that in its descent with the pelvis, the additional bulk given by it to the breech and body, so dilates the passages as to admit of the more easy and rapid exit of the head, and consequently lessons the danger of pressure upon the cord. However plausible this may appear, one thing is certain, that had the old plan of seizing both feet been observed in this instance, the result would in all probability have been different, for there would have been no remaining foot to prevent, by its malposition, the ascent of the head and complete version taking place. The woman had no untoward symptoms and was discharged on the tenth day.

CASE 2d.—Maria Briget, aged 25—first labour—presented herself at the Hospital on the morning of the 13th of September, stating that about 55 or 60 hours previous, she was delivered of a child in the country, seven miles from the city, and that her attendant discovering the existence of a second in the uterus and not knowing what to do, placed her in a cab for the Rotunda Hospital. After her admission into the Lying-In ward, an examination verified her statement as to there being a second child; the cord of the one of which she was delivered in the country was hanging from the vagina, the membranes were intact, the os dilatable and dilated to the size of a half crown. Close examination revealed a half breech presentation with what was supposed to be the edge of a placenta, barely in reach of the finger. There appeared to be no necessity for immediate interference and she was allowed to remain in this condition, (as there were occasional pains) for a more complete dilatation of the os uteri. In about an hour's time, the pains having gradually increased, she was delivered of a living child of about the eighth month development. The uterus remaining large, the finger was introduced and the breech presentation of a third child ascertained. Just at this moment profuse hæmorrhage commenced, and as there was no uterine action and the woman already debilitated, the hand was pushed up, the feet seized, and the labor rapidly terminated. The irritation made upon the womb by introducing the hand, excited sufficient contractions to arrest the hæmorrhage for 10 or 15 minutes, but it soon occurred again, and as the placentæ could not be extruded either by traction at the cord or pressure upon the uterus, the hand had a second time to be introduced to bring them away, this was readily accomplished notwithstanding they were both morbidly adherent; the hæmorrhage immediately abated and was soon entirely arrested under the application of cold, and the administration of ergot, brandy and cold stimulating injections, per rectum, thrown by a long elastic tube for the purpose, high up in the bowels, but all was to no avail, for she expired in spite of all effort in about an hour from the extraction of the placentæ, her death resulting apparently not so much from loss of the vital fluid as from the great shock to the system generally.

The great error committed in the management of this case, was the omission of the gentleman who attended her in the country, to complete the labour by rupturing the membranes of the children in utero, soon after the birth of the first. The hæmorrhage and retention of the placentæ could not it is true, have been forseen nor perhaps obviated even by him, but her system would certainly then have better borne the shock induced by the rough manipulations required by the circumstances, than after the 50 or 60 hours which elapsed between the birth of the first and second child, during the whole of which time she was probably being enfeebled by the slow hæmorrhage which must have resulted from the partial detachment of one or both placentæ, and especially of the one of the first child, which was attached so near the cervix as almost to make it a case of partial placentæ previa. Sept. 14th Autopsy.

During the act of, and after parturition, there was discovered from its inordinate size and firmness a morbid growth of the uterus which led to an inquiry as to its true character. The Post Mortem examination disclosed a large fibrous tumor of near three pounds weight, occupying the whole anterior half of the organ. Doubtless this tumor exerted some influence in the occurrence of premature labour, and had also perhaps some agency in the production of the unfortunate complications of hæmorrhage, retained placentæ, &c. But it is interesting to observe how, under such circumstances, nature will so accomplish her physiological functions of conception, gestation, and though but imperfectly, even that of parturition.

P. S.—There are also other cases of interest which I may send you, should time permit.

Your friend &c.,

WM. I. ARRINGTON.

---

